# Single-nucleotide polymorphism typing analysis for molecular subtyping of *Salmonella* Tennessee isolates associated with the 2007 nationwide peanut butter outbreak in the United States

**DOI:** 10.1186/s13099-017-0176-y

**Published:** 2017-05-01

**Authors:** Hee-Jin Dong, Seongbeom Cho, David Boxrud, Shelly Rankin, Francis Downe, Judith Lovchik, Jim Gibson, Matt Erdman, A. Mahdi Saeed

**Affiliations:** 1 BK21 PLUS Program for Creative Veterinary Science Research, Research Institute for Veterinary Science and College of Veterinary Medicine, Seoul National University, Seoul, 08826 South Korea; 2 Minnesota Department of Health, St Paul, MN 55164 USA; 3 Department of Microbiology, School of Veterinary Medicine, University of Pennsylvania, Kennett Square, PA 19348 USA; 4 Michigan Department of Health, Lansing, MI 48909 USA; 5 Indiana State Department of Health, Indianapolis, IN 46204 USA; 6 Tennessee Department of Health, Nashville, TN 37247 USA; 7 NVSL USDA, National Veterinary, Services Laboratories, a unit within the U.S. Department of Agriculture, Riverdale, MD 20737 USA; 8 Departments of Large Animal Clinical Sciences and Epidemiology and Biostatistics, Michigan State University, East Lansing, MI 48824 USA

**Keywords:** *S*. Tennessee, SNP typing, Peanut butter outbreak

## Abstract

**Background:**

In 2007, a nationwide *Salmonella* Tennessee outbreak occurred via contaminated peanut butter. Here, we developed a single-nucleotide polymorphism (SNP)-typing method for *S*. Tennessee to determine the clonal subtypes of *S*. Tennessee that were associated with the peanut butter outbreak.

**Methods and results:**

One seventy-six *S*. Tennessee isolates from various sources, including humans, animals, food, and the environment, were analyzed by using the SNP technique. Eighty-four representative SNP markers were selected by comparing the sequences of three representative *S*. Tennessee strains with different multi-locus sequence typing and variable number tandem repeats from our collection. The set of eighty-four SNP markers showed 100% typeability for the 176 strains, with the nucleotide diversity ranging from 0.011 to 0.107 (mean = 0.049 ± 0.018, median = 0.044) for each marker. Among the four clades and nine subtypes generated by the SNP typing, subtype 1, which comprised 142 *S*. Tennessee strains, was the most predominant. The dominance of single-strain clones in subtype 1 revealed that *S*. Tennessee is highly clonal regardless of outbreak-association, source, or period of isolation, suggesting the presence of an *S*. Tennessee strain prototype. Notably, a minimum 18 SNP set was able to determine clonal *S*. Tennessee strains with similar discrimination power, potentially allowing more rapid and economic strain genotyping for both outbreaks and sporadic cases.

**Conclusions:**

The SNP-typing method described here might aid the investigation of the epidemiology and microevolution of pathogenic bacteria by discriminating between outbreak-related and sporadic clinical cases. In addition, this approach enables us to understand the population structure of the bacterial subtypes involved in the outbreak.

**Electronic supplementary material:**

The online version of this article (doi:10.1186/s13099-017-0176-y) contains supplementary material, which is available to authorized users.

## Background

*Salmonella* is a bacterial pathogen that causes foodborne illnesses worldwide. It is estimated that more than 1.2 million cases of salmonellosis are reported in the United States annually, resulting in 23,000 and 450 cases of hospitalization and death, respectively [1]. Among the 2500 serotypes of *Salmonella* spp., *S*. Tennessee is rarely isolated and is responsible for <0.1% of *Salmonella* infections [2]. However, in 2006–2007, a large and nationwide outbreak of *S.* Tennessee infections occurred in the United States, and the consumption of contaminated peanut butter was found to be strongly associated with this outbreak [3, 4]. The outbreak lasted for over a year, leading to approximately 715 reported cases in 48 states [5]. While most infected people had gastrointestinal symptoms, including diarrhea, fever, and abdominal pain, more than a third of them had a urinary tract infection [4, 5]. Urinary tract infection caused by *Salmonella* species is rare, and some researchers presumed that this may be related to the prolonged survival of *S*. Tennessee in the environment, highlighting the necessity of molecular subtyping to detect outbreak-related strains from the environmental background [5, 6]. Several studies have revealed the high virulence and survivability of *S*. Tennessee strains [7–10]. In addition, because peanut butter has a long shelf life, contamination might result in *S*. Tennessee infections over the long term. *S*. Tennessee was identified from unopened peanut butter during another peanut butter outbreak caused by *S*. Typhimurium in 2009, indicating that sporadic cases of *S*. Tennessee infection may have occurred upon the consumption of contaminated peanut butter by individuals who did not know of the peanut butter outbreak [11].

Several molecular-based techniques are used to differentiate and identify the relatedness of *Salmonella* species. Pulsed-field gel electrophoresis (PFGE), a well-known molecular typing method, has been used as the “gold standard” for subtyping *Salmonella* spp. The peanut butter outbreak-associated *S*. Tennessee strains have the unique CDC PulseNet PFGE profiles of *Xba*I patterns JNXX01.0010, JNXX01.00011, and JNXX01.0026, which were used to determine their association with this outbreak [5]. However, PFGE is a labor-intensive technique requiring more than 2 days to perform. In addition, the PFGE technique does not always optimally discriminate the bacterial strains, especially closely related strains [12]. To overcome these disadvantages, several molecular subtyping methods, including multi-locus variable-number tandem repeat analysis (MLVA) or multi-locus sequence typing (MLST), were adapted for differentiating *Salmonella* serovars [13, 14]. Despite the many advantages of these techniques, MLVA was found to be less effective for long-term epidemiological studies owing to the instability of some loci that evolve quickly [15, 16]; furthermore, the usefulness of MLST for the investigation of outbreaks is controversial owing to the limited number of mutations within the housekeeping genes used for the MLST study [17, 18]. As an alternative technique, a single-nucleotide polymorphism (SNP) method was introduced. SNPs located in the bacterial genome, and selection of multiple loci from genes with high polymorphism, including genes associated with quinolone resistance or flagella antigen, can be used to discriminate the genetic relatedness in a bacterial population and trace the evolutionary origin of a bacterial species. With this advantage, the SNP-typing method is often used to investigate the epidemiology of an outbreak and the mutational events for tracing the temporal and geographical origin of particular bacteria [12, 18]. To date, only a few SNP-typing methods have been developed for *Salmonella* spp. [19–21]. The development of novel SNP-typing tools would play an important role in identifying unrelated stains of *Salmonella* spp. [12].

In this study, an SNP-typing method was developed for *S*. Tennessee to determine the clonal subtypes of *S*. Tennessee that were associated with the peanut butter outbreak. In addition, SNP markers were applied to isolates in order to evaluate the genetic relatedness of *S*. Tennessee strains isolated from various sources. Finally, the minimum set of SNP markers required to determine clonal *S*. Tennessee strains more rapidly and cost-effectively was identified.

## Methods

### Procurement of *S.* Tennessee strains and epidemiological data

A total of 176 *S*. Tennessee isolates from humans, animals, food, and the environment were procured from eight institutes located in Minnesota, Michigan, Indiana, Tennessee, New York, Iowa, Pennsylvania, and Calgary (Canada). Of the *S.* Tennessee isolates, 131 were obtained from five state Departments of Health in the United States, and epidemiological data, including age, sex, isolation date, and PFGE results, were collected for the human isolates, when available. Forty-five *S*. Tennessee stains from diverse animal and environmental sources were procured from three institutions (University of Pennsylvania, *Salmonella* Reference Center; University of Calgary, *Salmonella* Genetic Stock Center; and the National Veterinary Service Laboratory, Ames). Outbreak-associated *S*. Tennessee stains were defined as those causing onset of illness or isolation during the period from Aug, 01, 2006 to Jul, 31, 2007, and having PFGE profiles of JNXX01.0010, JNXX01.0011, or JNXX01.0026 [5] (Table 1).

**Table 1:** Information of strains used in this study

Source	Site of isolation	Location	Outbreak_range^a^	Outbreak_PFGE^b^	Outbreak association^c^
Human (114)^d^	Stool (60)	IN (7), MI (17), MN (34), NY (19), TN (37)	Yes (81)	Yes (67)	Yes (64)
	Urine (32)		No (32)	No (7)	Suspected (20)
	Wound (2)		Unknown (1)	Unknown (40)	No (30)
	Unknown (20)				
Food (17)	Peanut butter (7)	MN (13), TN (2), UC (2)	Yes (13)	Yes (9)	Yes (7)
	Dried powdered eggs (6)		No (2)	Unknown (8)	Suspected (8)
	Ground beef (1)		Unknown (2)		No (2)
	Fish meal (1)				
	Unknown (2)				
Environment (8)	Feed (1)	MN (2), UP (6)	Yes (2)	No (2)	No (8)
			Unknown (6)	Unknown (6)	
	Unknown (7)				
Animal (37)	Avian (24); chicken, chukar, pheasant, turkey, etc.	NVSL (23), UP (14)	Unknown (37)	Unknown (37)	No (37)
	Ruminant (10); alpaca, cattle, deer, goat				
	Swine (3)				

*IN* Indiana, *MN* Minnesota, *MI* Michigan, *NVSL* National Veterinary Service Laboratory, *NY* New York, *TN* Tennessee, *UC* Salmonella genetic stock center at the University of Calgary, *UP* Salmonella reference center at the University of Pennsylvania

^a^Outbreak_range: yes, illness onset or isolation of *S.* Tennessee during 2006.08.01 to 2007.07.31; no, illness onset or isolation of *S*. Tennessee before 2006.08.01 or after 2007.07.31; unknown, no information on illness onset or isolation date

^b^Outbreak_PFGE: yes, PFGE profiles of JNXX01.0010, JNXX01.0011, or JNXX01.0026; no, PFGE profiles other than JNXX01.0010, JNXX01.0011, or JNXX01.0026; unknown, no PFGE profile data

^c^Outbreak association: yes, both yes for outbreak range and PFGE; suspected, yes for either outbreak range or PFGE; no, both no and/or unknown for outbreak range and PFGE

^d^Numbers in parentheses indicate the number of isolates

### Selection of representative strains from various sources for the identification SNP markers

To select epidemiologically diverse *S*. Tennessee strains from humans, animals, food, and the environment, 60 isolates of *S.* Tennessee were selected based on diverse PFGE patterns and unrelated epidemiologic information considering factors such as time of isolation and source. These selected isolates were then further screened by using MLST and VNTR as described below to select representative *S*. Tennessee strains. MLST was performed on seven housekeeping genes, *thrA, purE*, *sucA*, *hisD*, *aroC*, *hemD*, and *dnaN*, which were derived from the *Salmonella* MLST database (http://mlst.warwick.ac.uk/mlst/dbs/Senterica). Phylogenetic analysis was performed by pairwise comparison of the nucleotide sequences of these seven MLST genes to illustrate the neighbor-joining tree. For the VNTR analysis, tandem repeats of locus SE5 were analyzed using previously designed primers [14].

### Identification of SNPs

To identify SNPs, the sequences of three representative *S*. Tennessee strains, MN25, TN32, and MN47 were compared. The three strains, which represented different MLST and VNTR types, were selected from 60 diverse *S*. Tennessee strains. The genotypic and epidemiologic features of the three strains were as follows: (i) MN25: outbreak-associated strain, isolated from peanut butter during Feb 2007, PFGE pattern of JNXX01.0011, major MLST type, and allele 14 by VNTR; (ii) TN32: outbreak-associated strain, isolated from patient urine during Mar 2007, PFGE pattern of JNXX01.0026, major MLST type, and allele 13 by VNTR; and (iii) MN47: non-outbreak-associated strain, isolated from patient stool during Jan 2008, PFGE pattern of JNXX01.0049, minor MLST type, and allele 8 by VNTR.

Purified DNA from a strain isolated from peanut butter was submitted to the Genomic Core of the Research Technology Support Facility (RTSF) at Michigan State University for pyrosequencing using the 454 GS-FLX Titanium platform. Genome assembly of the produced data identified 66 gaps (range 0.2–1.8 kb) within 14 scaffolds that covered 4.8 Mb of the genome. Assembled sequences were deposited in the Genome Project database (NCBI accession number: PRJNA 46571).

The sequences of three representative strains, MN25, TN32, and MN47 were compared. Two sequence sets, MN25 from this study and the *S*. Tennessee strain CDC07-0191, were aligned using the NUCmer version 3.07 alignment program [22], which revealed that the two strains were nearly identical at the genomic level, having <0.005% (1/20,000) SNPs. The shotgun reads from TN32 and MN47 were then aligned to the MN25 sequence using the Roche 454/GS Reference Mapper program, version 2.0.01.14 (Madison, WI, USA). Putative SNPs were generated by comparison of the consensus contigs to the reference genome (MN25).

### Application of SNP typing methods to the clinical isolates

The newly detected SNP markers were applied to the human, animal, food, and environmental isolates for evolutionary and molecular epidemiological analyses. The nucleotide diversity (pi, *π*) was calculated using Nei’s diversity index to measure the degree of polymorphism of each marker within the *S*. Tennessee isolates. A phylogenetic dendrogram for SNP subtypes was computed by using the unweighted pair group method with arithmetic mean (UPGMA) analysis for categorical value, and a minimum spanning tree (MST) was constructed using BioNumerics version 6.6 (Applied Maths NV, Belgium). To identify the minimal set of SNP markers required to determine clonal *S*. Tennessee strains, SNP markers having higher nucleotide diversity (*π* > 0.09) were first selected, and then representative markers (minimum SNP set) were randomly selected from among a set of markers with the same profile. The MST was constructed for the 176 isolates with this minimum SNP set.

## Results

### Identification of SNP markers

Markers were identified based on the comparison of three representative *S*. Tennessee strains, MN25, TN32, and MN47. Among a total of 16,221 SNPs identified, 2630 (16.2%) non-synonymous SNPs (nsSNPs) and 13,591 (83.8%) synonymous SNPs (sSNPs) were identified. Of them, SNPs that did not have other SNPs in their surroundings (within 50 base pairs in both directions) were selected from intragenic regions of the genome sequences, and SNPs that were found to be singletons when applied to 176 isolates of *S*. Tennessee were excluded. Finally, 84 SNP markers were selected from the 16,221 SNPs. Among the selected markers, 57 (67.9%) were sSNPs and 27 (32.1%) were nsSNPs. The 84 SNPs were allocated within 61 genes, each of which contained 1–5 SNPs (Table 2; Additional file 1: Table S1).

**Table 2:** SNP profiles for *S.* Tennessee strains

Clade	Subtype	No. of isolates	SNP profiles										
			1	2	3	4	5	6	7	8	9	10	11
1	Subtype1	142	C	A	G	G	T	G	C	C	G	C	G
	Subtype2	8	•	•	•	•	•	•	•	•	•	•	•
	Subtype3	9	•	•	•	•	•	•	•	•	•	•	•
	Subtype4	10	•	•	•	•	•	•	•	•	•	•	•
	Subtype5	1	•	•	•	•	•	•	•	•	•	•	•
2	Subtype6	2	•	C	•	•	C	A	•	•	•	T	•
3	Subtype7	1	•	C	•	•	C	•	•	T	•	T	•
4	Subtype8	1	T	C	A	A	C	A	T	T	•	T	C
	Subtype9	2	T	C	A	A	C	A	T	T	A	T	C
Types^a^			S	S	N	S	S	N	S	S	N	S	S
Minimal set of SNPs^b^							*	*	*				

Clade	Subtype	No. of isolates	SNP profiles										
			12	13	14	15	16	17	18	19	20	21	22
1	Subtype1	142	G	A	A	A	G	T	A	T	G	G	A
	Subtype2	8	•	•	•	•	•	•	•	•	•	•	•
	Subtype3	9	•	•	•	•	•	•	•	•	•	•	•
	Subtype4	10	•	•	•	•	•	•	•	•	•	•	•
	Subtype5	1	•	•	•	•	•	•	•	•	•	•	•
2	Subtype6	2	•	G	•	G	•	C	•	•	•	•	C
3	Subtype7	1	•	G	•	G	•	C	•	C	•	•	C
4	Subtype8	1	A	G	G	G	A	C	C	C	A	A	C
	Subtype9	2	A	G	G	G	A	C	C	C	A	A	C
Types^a^			N	S	S	S	S	S	N	S	S	S	N
Minimal set of SNPs^b^								*				*	

Clade	Subtype	No. of isolates	SNP profiles											
			23	24	25	26	27	28	29	30	31	32	33	34
1	Subtype1	142	T	A	T	T	T	C	G	A	C	T	T	C
	Subtype2	8	•	•	•	•	•	•	•	•	•	•	•	•
	Subtype3	9	•	•	•	•	•	•	•	•	•	•	•	•
	Subtype4	10	•	•	•	•	•	•	•	•	•	•	•	•
	Subtype5	1	•	•	•	•	•	•	•	•	•	•	•	•
2	Subtype6	2	C	•	C	•	•	A	•	G	•	C	C	•
3	Subtype7	1	C	•	•	•	C	A	•	G	•	C	C	•
4	Subtype8	1	C	G	C	C	C	A	A	G	T	C	C	A
	Subtype9	2	C	G	C	C	C	A	A	G	T	C	C	A
Types^a^			S	S	S	N	S	S	N	S	S	S	N	S
Minimal set of SNPs^b^												*		

Clade	Subtype	No. of isolates	SNP profiles										
			35	36	37	38	39	40	41	42	43	44	45
1	Subtype1	142	C	G	C	G	A	C	A	T	T	T	T
	Subtype2	8	•	•	•	•	•	•	•	•	•	•	•
	Subtype3	9	•	•	•	•	•	•	•	•	•	•	•
	Subtype4	10	•	•	•	•	•	•	•	•	•	•	•
	Subtype5	1	•	•	•	•	•	•	•	•	•	•	•
2	Subtype6	2	T	A	•	•	•	•	G	C	C	•	C
3	Subtype7	1	•	A	•	•	•	•	G	•	C	C	C
4	Subtype8	1	T	A	T	A	T	T	G	C	C	C	C
	Subtype9	2	T	A	T	A	T	T	G	C	C	C	C
Types^a^			S	S	N	S	S	N	N	S	N	S	S
Minimal set of SNPs^b^				*	*								*

Clade	Subtype	No. of isolates	SNP profiles										
			46	47	48	49	50	51	52	53	54	55	56
1	Subtype1	142	T	C	C	A	A	G	C	A	A	A	G
	Subtype2	8	•	•	•	•	•	•	•	•	•	•	•
	Subtype3	9	•	•	•	•	•	•	•	•	•	•	•
	Subtype4	10	•	•	•	•	•	•	•	•	•	•	•
	Subtype5	1	•	•	•	•	•	•	•	•	•	•	•
2	Subtype6	2	C	•	•	G	G	A	•	•	G	•	•
3	Subtype7	1	C	•	•	G	G	A	•	•	G	G	A
4	Subtype8	1	C	T	T	G	G	A	T	G	G	G	A
	Subtype9	2	C	T	T	G	G	A	T	G	G	G	A
Types^a^			S	N	N	S	S	S	S	S	N	N	S
Minimal set of SNPs^b^									*				

Clade	Subtype	No. of isolates	SNP profiles										
			57	58	59	60	61	62	63	64	65	66	67
1	Subtype1	142	C	C	A	A	T	G	C	C	G	T	G
	Subtype2	8	•	•	•	•	•	•	•	•	•	•	•
	Subtype3	9	•	•	•	•	•	•	•	•	•	•	•
	Subtype4	10	•	•	•	•	•	•	•	•	•	•	•
	Subtype5	1	•	•	•	•	•	•	•	•	•	•	•
2	Subtype6	2	•	•	G	G	•	•	•	•	A	C	•
3	Subtype7	1	•	•	G	G	•	•	•	•	A	•	•
4	Subtype8	1	G	T	G	G	C	•	T	T	A	C	A
	Subtype9	2	G	T	G	G	C	T	T	T	A	C	A
Types^a^			N	S	S	N	S	N	S	S	S	S	N
Minimal set of SNPs^b^			*								*		*

Clade	Subtype	No. of isolates	SNP profiles										
			68	69	70	71	72	73	74	75	76	77	78
1	Subtype1	142	A	G	C	G	T	T	A	G	C	G	C
	Subtype2	8	•	•	•	•	•	•	•	•	•	•	•
	Subtype3	9	•	•	•	•	•	•	•	•	•	•	•
	Subtype4	10	•	•	•	•	•	•	•	•	•	•	•
	Subtype5	1	•	•	•	•	•	•	•	•	•	•	•
2	Subtype6	2	G	•	•	A	C	C	•	•	•	•	T
3	Subtype7	1	•	•	•	A	C	C	G	A	•	•	•
4	Subtype8	1	G	A	T	A	C	C	G	A	T	A	T
	Subtype9	2	G	A	T	A	C	C	G	A	T	A	T
Types^a^			S	S	N	N	S	S	N	S	N	S	S
Minimal set of SNPs^b^										*			

Clade	Subtype	No. of isolates	SNP profiles					
			79	80	81	82	83	84
1	Subtype1	142	C	C	C	C	G	T
	Subtype2	8	•	•	A	•	•	•
	Subtype3	9	•	•	•	•	C	•
	Subtype4	10	•	•	•	T	•	•
	Subtype5	1	•	•	A	•	•	A
2	Subtype6	2	•	•	•	•	•	•
3	Subtype7	1	T	•	•	•	•	•
4	Subtype8	1	T	T	•	•	•	•
	Subtype9	2	T	T	•	•	•	•
Types^a^			S	S	S	S	N	N
Minimal set of SNPs^b^				*	*	*	*	

^a^Types of SNPs: *N* non-synonymous, *S* synonymous

^b^Minimal set of SNP markers that can subtype the *S*. Tennessee strains used in this study

### Application of the SNP-typing method to *S.* Tennessee isolates from multiple sources

A total of 176 *S*. Tennessee isolates consisting of 114 human, 17 food, 8 environmental, and 37 animal isolates were obtained. Of these, 71 strains were found to be associated with the peanut butter outbreak. Among the 105 strains that were not matched to our definition of outbreak association, 28 strains were classified as a suspect group, as they contained strains either isolated during the defined period without having the designated PFGE profile or those that exhibited the defined PFGE profile with unknown isolation period (Table 1).

**Fig. 1:**
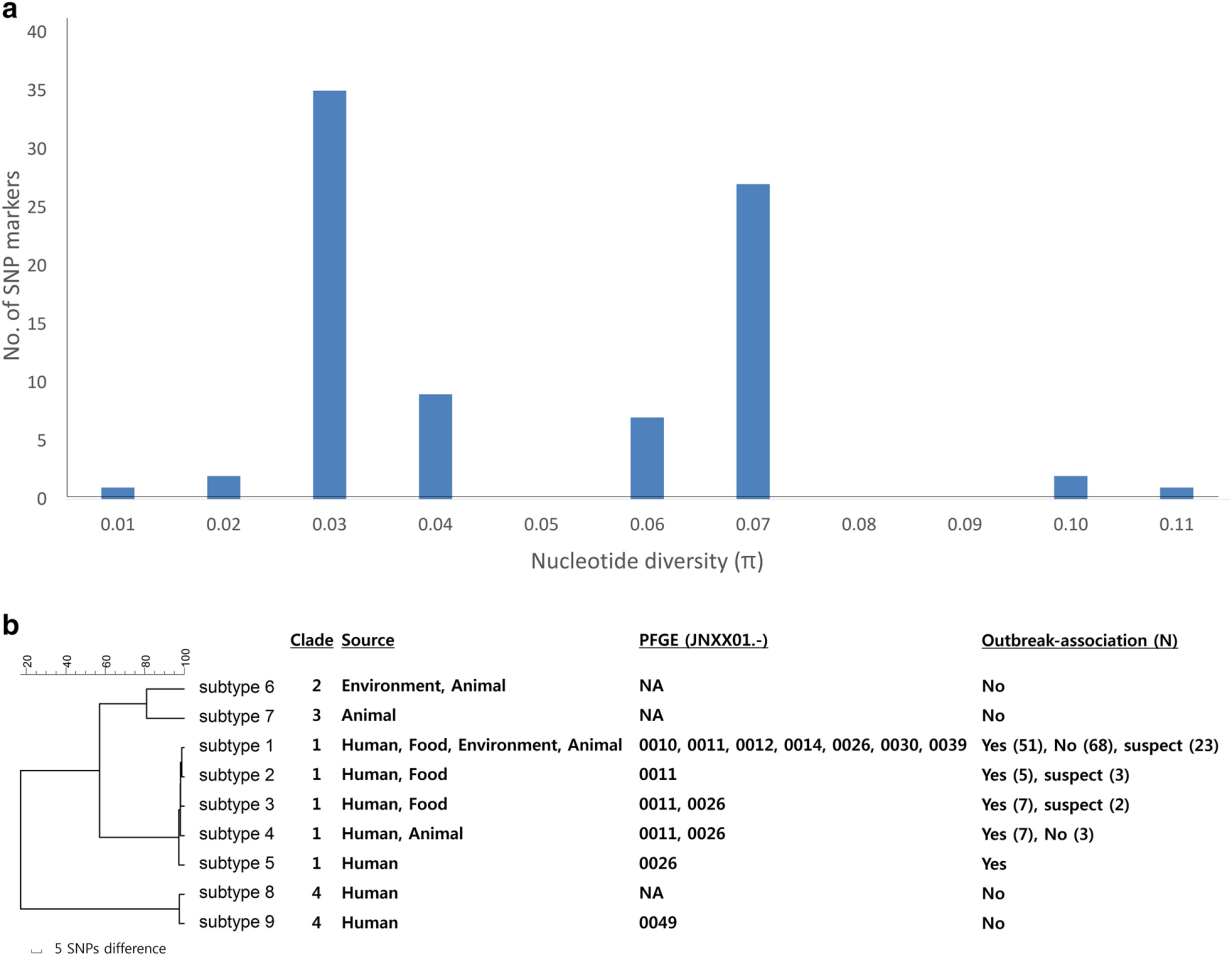
Distribution of 84 single-nucleotide polymorphisms (SNP) markers and their phylogenetic relationships. **a** Nucleotide diversity of the 84 SNP markers ranged from 0.011 to 0.107 (mean = 0.049 ± 0.018, median = 0.044). **b** The 84 SNP markers were able to type 176 *Salmonella* Tennessee strains, showing 100% typeability. The phylogenetic relationships of the 176 *S*. Tennessee strains were generated using the unweighted pair group method with arithmetic mean (UPGMA), which identified four clades and nine subtypes. *Outbreak association: yes; illness onset or isolation of *S*. Tennessee during 2006.08.01 to 2007.07.31, no; illness onset or isolation of *S*. Tennessee before 2006.08.01 or after 2007.07.31, suspect: strains that were isolated during 2006.08.01 to 2007.07.31 with unknown PFGE results or with PFGE profiles of JNXX01.0010, JNXX01.0011, or JNXX01.0026 with an unknown date of isolation or illness onset. Numbers in parenthesis indicate the number of isolates. NA: PFGE profile not available

SNP typing was performed using 84 SNP loci for 176 *S*. Tennessee isolates, demonstrating 100% typeability for the SNP method. The nucleotide diversity (*π*) of each of the 84 SNP markers ranged from 0.011 to 0.107 (mean = 0.049 ± 0.018, median = 0.044). Of the 84 SNP markers, one (marker number 84) was found to be a singleton that showed the lowest nucleotide diversity (*π* = 0.011), while another (marker number 82) had a maximum nucleotide diversity of 0.107 (Fig. 1a). The 84 SNPs categorized the 176 isolates into nine subtypes, which were clustered into four clades (Table 2; Fig. 1b). Clade 1 was the most predominant and included 170 isolates (96.6%) that belonged to subtypes 1–5. Among the subtypes, subtype 1 was found to be the most predominant subtype, comprising 142 isolates (80.7%).

A minimum SNP set was designed to determine clonal *S*. Tennessee strains more effectively. To this end, 18 SNP markers were selected, and this minimum set of 18 SNP markers was able to classify the 176 strains into four clusters and seven subtypes (Additional file 2: Figure S1).

### Relationship between SNP genotypes and isolates

**Fig. 2:**
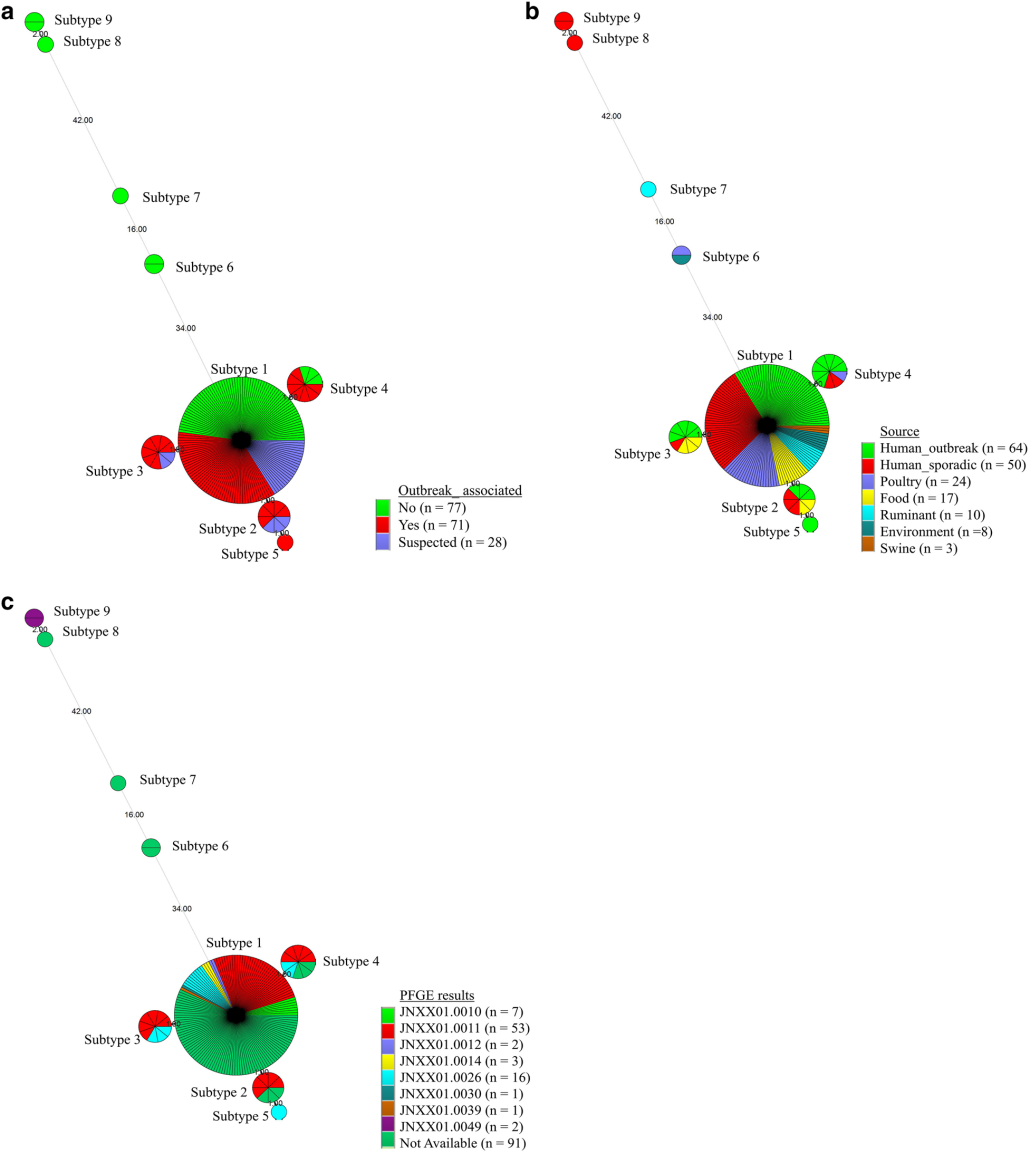
Minimum spanning trees of 176 isolates based on 84 SNP markers. Epidemic relationships of SNP profiles were generated via minimum spanning tree (MST) analysis using Bionumeric, version 6.6. Each *circle* represents a subtype; their sizes are proportionate to the number of isolates in each subtype. The length of the* line* connecting each* circle* is proportionate to the number of SNP markers that differ from each other. The relationships between the isolates and outbreak (**a**) association, (**b**) source, and (**c**) PFGE results are shown

The relationship between the genotypes and isolates was investigated based on the SNP typing results and the epidemiological data collected from various geographical locations. The relationships were visualized by MST to show the evolutionary distance between the isolates (Fig. 2).

All the outbreak-associated isolates were categorized into clade 1. Of the 71 outbreak-associated isolates, 51 (71.8%) belonged to subtype 1, while 20 (28.2%) isolates belonged to subtypes 2, 3, 4, and 5. In subtypes 2–5, the SNP profiles only differed by one or two markers from subtype 1, and most isolates were associated or suspected to be associated with the peanut butter outbreak, except for three isolates (two human isolates [NY04 and NY06] and one avian isolate [US15]). In subtype 1, 51 (35.9%), 23 (16.2%), and 68 (47.9%) outbreak-associated, suspected, and non-outbreak-associated isolates were included. While the sources of the outbreak-associated and suspected isolates were either humans or food, non-outbreak-associated strains were isolated from various sources, including humans (25; 36.8%), animals (34; 50.0%), food (2; 2.9%), and the environment (7; 10.3%; Fig. 2a, b).

In clades 2–4, six strains were included. The SNP profiles of these six strains differed by 48–80 markers from that of subtype 1. Of the six strains, three were isolated from humans (MN46, MN47, and NY01) whereas the other three strains were isolated from animals (UP16 from chicken and US17 from alpaca) or the environment (UP09; Fig. 2b).

Among 85 isolates with known PFGE profiles, 76 (89.4%) had *S*. Tennessee outbreak-related PFGE profiles (JNXX01.0010, JNXX01.0011, and JNXX01.0026) as determined by CDC PulseNet. All 76 isolates belonged to subtypes 1, 2, 3, 4, and 5 in clade 1, consistent with the SNP results. However, strains in subtypes 2 and 5 showed a single PFGE profile, and strains in subtypes 3 and 4 exhibited the PFGE profile of either JIXX01.0011 or JIXX01.0026. In addition, strains in subtype 1 had the most variable PFGE profile, with seven different profiles (JNXX01.0010, JNXX01.0011, JNXX01.0012, JNXX01.0014, JNXX01.0026, JNXX01.0030, and JNXX01.0039; Fig. 2c).

## Discussion

Prior to 2006, *S*. Tennessee was not a common *Salmonella* serovar, resulting in a relatively small number of *S*. Tennessee infections worldwide. Only one outbreak of *S*. Tennessee infection was reported to the United States (US) Centers for Disease Control associated with contaminated powdered milk products and infant formula [23]; in contrast, most cases of *S*. Tennessee infection were sporadic with unknown sources. However, after the multistate peanut butter outbreak of *S*. Tennessee in the US, several *S*. Tennessee-related outbreaks have occurred in humans, animals, and environments, revealing the persistent contamination of *S*. Tennessee strains across various sources [24–26]. In addition, a recent report on the association of *S*. Tennessee infection between babies and reptiles highlights the importance of *S*. Tennessee as a zoonotic pathogen [26]. To cope with the increase of *S*. Tennessee infection cases, an SNP-typing method was developed to evaluate the epidemiology of the peanut butter outbreak, and ultimately, to identify the mutational events of *S*. Tennessee strains.

The comparison of three representative *S*. Tennessee strains identified numerous SNPs, most of which were sSNPs. While synonymous mutations are considered as being neutral, causing minimal effect on the organisms, non-synonymous mutations sometimes lead to functional changes that may provide a positive selection for the pathogen toward spreading infections [27, 28]. Some nsSNPs were found to be associated with bacterial colonization or host specificity [28, 29]. In this study, one SNP marker (marker number 9) was found to be an nsSNP that replaced the amino acid glutamine with a stop codon. This marker is allocated within *omp*C, which encodes a major outer membrane protein. In a previous study, it was found that *omp*C was genetically stable in all tested *Salmonella* serotypes except *S*. Arizonae [30]. However, this SNP was observed in two *S.* Tennessee strains in the current study. While some studies have reported the detection of a higher proportion of sSNPs than nsSNPs [31], consistent with our study, the opposite phenomenon appears to be more common in highly clonal organisms [19, 32, 33]. Although the significance of this phenomenon has not yet been established [32, 34, 35], sSNPs remain useful markers for investigating the genetic characteristics required to trace evolutionary origin [12, 20].

Application of the 84 SNP markers (selected from three strains) for the comparison of the 176 *S*. Tennessee strain isolates revealed relatively low genetic diversity, with a mean nucleotide diversity of 0.049 ± 0.018, indicating that any two randomly selected isolates would differ by only 4.9% (Fig. 1). Generally, the nucleotide diversity of SNP markers is low, owing to the bi-allelic nature of SNP sites [36]. However, the nucleotide diversity in the current study was lower than our expectation, which might be due to sampling bias. A symmetrical sample collection is important to evaluate the discriminatory power for subtyping [21]. In the present study, the sample size was not sufficient for the evaluation of genetic diversity, because most of the human, food, and environmental samples were collected during or just after the peanut butter outbreak, which might cause the SNP analysis to not be representative of the entire spectrum of *S*. Tennessee strains. In addition, the high clonality of *Salmonella* spp. might contribute to lower genetic diversity. Minor genetic changes have been reported for *S*. Typhimurium DT41 by MLVA [37] and *S*. Tennessee by PFGE and MLST [38], indicating the overall genetic stability of *Salmonella* species.

Following our MST analysis, while all outbreak-associated strains were included in clade 1, some non-outbreak-associated strains were also included. In contrast to subtypes 2, 3, and 5, which consisted of outbreak-associated or outbreak-suspected strains, subtypes 1 and 4 consisted of outbreak and non-outbreak-associated strains. Considering that two strains in subtype 4 were isolated from humans (Dec 2007 and Nov 2007) shortly after the peanut butter outbreak during the period from Aug 2006 to Jul 2007, late infection by *S*. Tennessee outbreak-related strains might be possible. Non-outbreak-associated strains in subtype 1 mainly consisted of animal isolates. Although several *S*. Tennessee strains were isolated from animals during the peanut butter outbreak, the animal isolates used in this study did not include outbreak-associated strains. Notably, the CDC records showed that *S*. Tennessee isolates from chicken, porcine, and turkey sources were non-clinical, whereas bovine, turkey, other animals, and environmental sources were clinical, suggesting the possibility of chicken as an asymptomatic carrier of *S*. Tennessee strains [39]. In addition, two non-outbreak-associated strains in subtypes 1 and 4 were also isolated from poultry, implying a close relationship between the human and poultry isolates.

The results of the two subtyping methods, PFGE and SNP, were compared. While all the strains exhibiting the outbreak-related PFGE profile JNXX01.0010 belonged to subtype 1, the strains showing the PFGE profiles JNXX01.0011 and JNXX01.0026 belonged to a total of four and three subtypes, respectively, indicating the high discrimination power of the SNP typing method. On the other hand, subtypes 1, 3, and 4 consisted of strains with more than two kinds of PFGE profiles, indicating that neither method was sufficient to discriminate highly clonal *S*. Tennessee strains. Considering that single-nucleotide diversity at restriction enzyme sites results in three-band differences, one- or two-band differences among outbreak-related PFGE profiles suggest that the *S*. Tennessee strains are genetically stable [40].

Identification of minimal SNP marker sets can be beneficial for the rapid and economical determination of strain types. In the current study, a minimum set of 18 SNP markers was determined; these markers classified the 176 isolates into seven subtypes. While the 84 SNP markers generated nine subtypes, one marker that contributed to the generation of a subtype was found to be a singleton, and was excluded from the minimum set. Nevertheless, this minimum set of SNPs could likely be utilized to genotype *S*. Tennessee strains more rapidly and cost-effectively, and with similar discriminatory power as that of the complete 84 SNP panel.

Investigation of the outbreak of foodborne bacterial diseases using sequencing-based molecular typing is relatively new, and this approach will aid the investigation of the epidemiology and microevolution of pathogenic bacteria by discriminating between outbreak-related and sporadic clinical cases. In addition, this approach enables us to understand the population structure of the bacterial subtypes involved in the outbreak. While our method does not have direct applications in the clinical setting, we believe that this study would help identify the evolutionary origin of an outbreak.

## Conclusions

In conclusion, we developed, for the first time, an SNP-typing method for *S*. Tennessee strains and demonstrated that the sets of informative SNP markers were able to determine clonal *S*. Tennessee strains. The dominance of single clones of *S*. Tennessee strains in subtype 1 revealed that *S*. Tennessee is highly clonal, regardless of outbreak association, source, or period of isolation, suggesting the presence of an *S*. Tennessee strain prototype. Furthermore, a minimum set of SNP markers was identified that would likely provide advantages for genotyping *S*. Tennessee strains more rapidly and economically, especially during outbreaks or for sporadic cases. The SNP-typing method described here might also be useful for monitoring *S*. Tennessee strains to obtain a better understanding of their evolutionary dynamics. The continual monitoring of mutational events using *S*. Tennessee with this SNP-typing method might be an effective strategy for investigating the genetic relatedness of *S.* serovar Tennessee and to control and prevent *S*. Tennessee infections.

## Additional files

**Additional file 1: Table S1.** Sequences for 84 SNP markers.

**Additional file 2: Figure S1.** Minimum spanning trees of 176 isolates based on 18 SNP markers. Epidemic relationships of SNP profiles were generated via minimum spanning tree (MST) analysis using Bionumeric, version 6.6. Each circle represents a subtype, and their sizes are proportional to the number of isolates in the each subtype. The length of the line connecting each circle is proportionate to the number of SNP markers that differ from each other. The subtype numbers were matched to the subtype generated by using 84 SNPs. The relationships between the isolates and outbreak association are illustrated.

## Abbreviations

MLVA: multi-locus variable-number tandem repeat analysis
MLST: multi-locus sequence typing
MST: minimum spanning tree
PFGE: pulsed-field gel electrophoresis
SNP: single-nucleotide polymorphism
UPGMA: unweighted pair group method with arithmetic mean
VNTR: variable number tandem repeat
