# Supplementary figures and images for: Single-nucleotide polymorphism typing analysis for molecular subtyping of Salmonella Tennessee isolates associated with the 2007 nationwide peanut butter outbreak in the United States
Source: Gut Pathog. 2017 May 1;9:25. doi: 10.1186/s13099-017-0176-y (PMC5412032; doi:10.1186/s13099-017-0176-y)

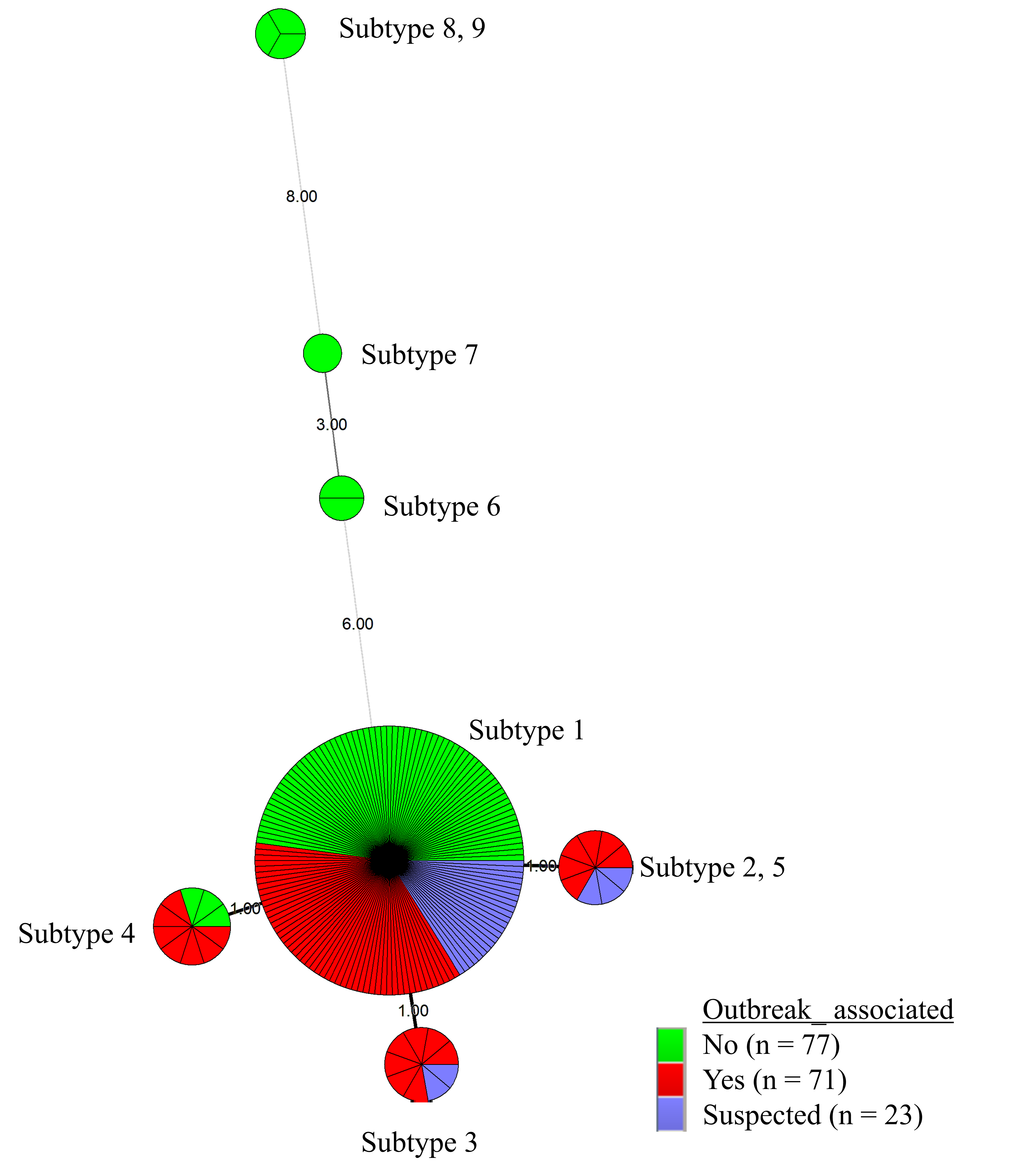

Supplement: Supplementary file 2 — Additional file 2: Figure S1. Minimum spanning trees of 176 isolates based on 18 SNP markers. Epidemic relationships of SNP profiles were generated via minimum spanning tree (MST) analysis using Bionumeric, version 6.6. Each circle represents a subtype, and their sizes are proportional to the number of isolates in the each subtype. The length of the line connecting each circle is proportionate to the number of SNP markers that differ from each other. The subtype numbers were matched to the subtype generated by using 84 SNPs. The relationships between the isolates and outbreak association are illustrated. [file 13099_2017_176_MOESM2_ESM.tif]
